# The influence of the steep medial posterior tibial slope on medial meniscus tears in adolescent patients: a retrospective case-control study

**DOI:** 10.1186/s12891-021-04766-9

**Published:** 2021-10-25

**Authors:** Xiangtian Deng, Hongzhi Hu, Qingcheng Song, Yiran Zhang, Weijian Liu, Lian Zhu, Yingze Zhang

**Affiliations:** 1 School of Medicine, Nankai University, Tianjin, 300071 People’s Republic of China; 2 Department of Orthopaedic Surgery of Hebei Province, Third Hospital of Hebei Medical University, 139 Ziqiang Road, Shijiazhuang, 050051 Hebei People’s Republic of China; 3 Department of Orthopedics, Union Hospital of Tongji Medical College of Huazhong University of Science and Technology, Wuhan, 430022 People’s Republic of China; 4 NHC Key Laboratory of Intelligent Orthopaedic Equipment, Third Hospital of Hebei Medical University, Shijiazhuang, 050051 Hebei People’s Republic of China

**Keywords:** Adolescent, Medial meniscal tears, Posterior tibial slope, Case-control study

## Abstract

**Background:**

Several studies have demonstrated a relationship between the posterior tibial slope (PTS) and meniscal tears in adults. However, little is known about the association between the PTS of the adolescents and medial meniscal tears (MMT). The purpose of this study was to evaluate the association between the PTS and MMT in adolescents, and to determine the optimal cut-off values of PTS for discriminating between the MMT and the control groups.

**Methods:**

Between January 2018 and January 2020, a retrospective case-control study was performed. In this study, isolated MMT adolescent patients with no ligamentous injuries were matched by age and sex to a control group of radiologically normal images. The PTS was defined as the angle between the perpendicular line to proximal tibial cortex (PTC) and the tangent line along the tibial plateau. Then, both the medial posterior tibial slope (MPTS) and lateral posterior tibial slope (LPTS) were measured by plain radiographs on the lateral views. In addition, the optimal cut-off values of PTS were determined by the receiver operating characteristic (ROC) curve analysis.

**Results:**

A total of seventy-two patients who met the inclusion criteria were enrolled in the final analysis (36 patients with isolated MMT, 36 controls). The MPTS was greater in the knees with isolated MMT (10.7° ± 2.1°) than that of the control group (8.8° ± 1.7°), showing significant difference (*P*<0.001). However, there was no significant difference regarding the LPTS between the isolated MMT and controls (11.5 ± 3.4 vs 10.9 ± 2.6, *p*>0.05). In the ROC curve analysis, the calculated cutoff value of the MPTS discriminating between the groups was 10.3°, with a sensitivity of 73.3% and specificity of 78.9%.

**Conclusions:**

This study demonstrated that steep MPTS is associated with MMT, and MPTS≥10.3° was identified to be a risk factor for MMT in adolescents.

## Background

Meniscal tears, occurred in adolescent patients, are common injuries concomitant with anterior cruciate ligament (ACL) ruptures during athletic activity. If left untread, meniscal tears are thought to carry potential sequelae, resulting in abnormal stress distribution within the tibiofemoral joint; therefore, accelerating the deterioration of the articular cartilage and early post-traumatic osteoarthritis [1, 2]. Overall, risk factors for meniscal tears are considered to be extrinsic and intrinsic. Extrinsic risk factors for meniscal tears is multifactorial, including neuromuscular forces, injury mechanisms, delay of surgery, untreated ligamentous injuries, and landing biomechanics during physical activity [3–5], while the intrinsic risk factors, including age, sex, lower limb alignment, high body mass index (BMI), and geometry of the tibial plateau [6–8]. Identification of these intrinsic anatomical factors, such as posterior tibial slope (PTS), may help orthopedic surgeons better recognize risk factors of meniscal tears in susceptible individuals.

In recent years, several studies have been conducted on the risk factors between the PTS and meniscal injuries in ACL-deficient knees [6, 8–12]. The bony morphology of the proximal tibia, especially a steep tibial slope, is known to have a negative effect on the biomechanics and kinematics of the knee [13, 14]. Furthermore, increased PTS is associated with the magnitude of the shear force in tibial platform and influences the biomechanics of the tibiofemoral joint, which may increase the risk of meniscal injuries in adults [10]. Actually, medial meniscal tears (MMT) are more frequently occurred due to the role in transmitting shear force with compressive force. Furthermore, medial meniscus also plays an important role in maintenance of knee stability [15–17]. Until now, however, potential anatomical risk factors for MMT in adolescents remain unclear. As is well known, meniscus is a critical regulator within the tibiofemoral joint with various functions, such as shock absorption, load diffusing, and enhancement of stability [18]. In addition, the medial meniscus is an important component that distributes compressive loading force across the knee and is subjected to anteroposterior shear forces. Walker et al. [19] reported that the medial meniscus delivers the higher percentage of shear force compared to lateral meniscus. Using knee radiographs and magnetic resonance imaging (MRI), Markl et al. [12] showed a statistically significant association between a steep PTS and MMT in adults, regardless of sex. Nevertheless, limitations of these studies are they focused on the relationship between the PTS and meniscal tears in adults. In clinical practice, it is also important for orthopedic surgeons to investigate the association between the PTS and MMT in adolescents.

Therefore, it is necessary to suppose that the morphology of the tibial may act as an important role in MMT because increased PTS would lead to higher shear force in the medial meniscus during compressive loading. To the best of our knowledge, however, there is a paucity of literatures regarding the relationship between the PTS and MMT in adolescents, despite their potential clinical importance. The purpose of this study was (a) to investigate the association between the PTS and MMT, and (b) to determine the optimal cut-off values of PTS for discriminating between the MMT and the control groups. It was hypothesized that an increased PTS may serve as a potential risk factor for MMT in this population.

## Methods

### Study design

This retrospective case-control study was performed at the Third Hospital of Hebei Medical University and approved by the local institutional ethics committee. All included patients provided written informed consent and this study complied with the principles of the Declaration of Helsinki.

**Fig. 1:**
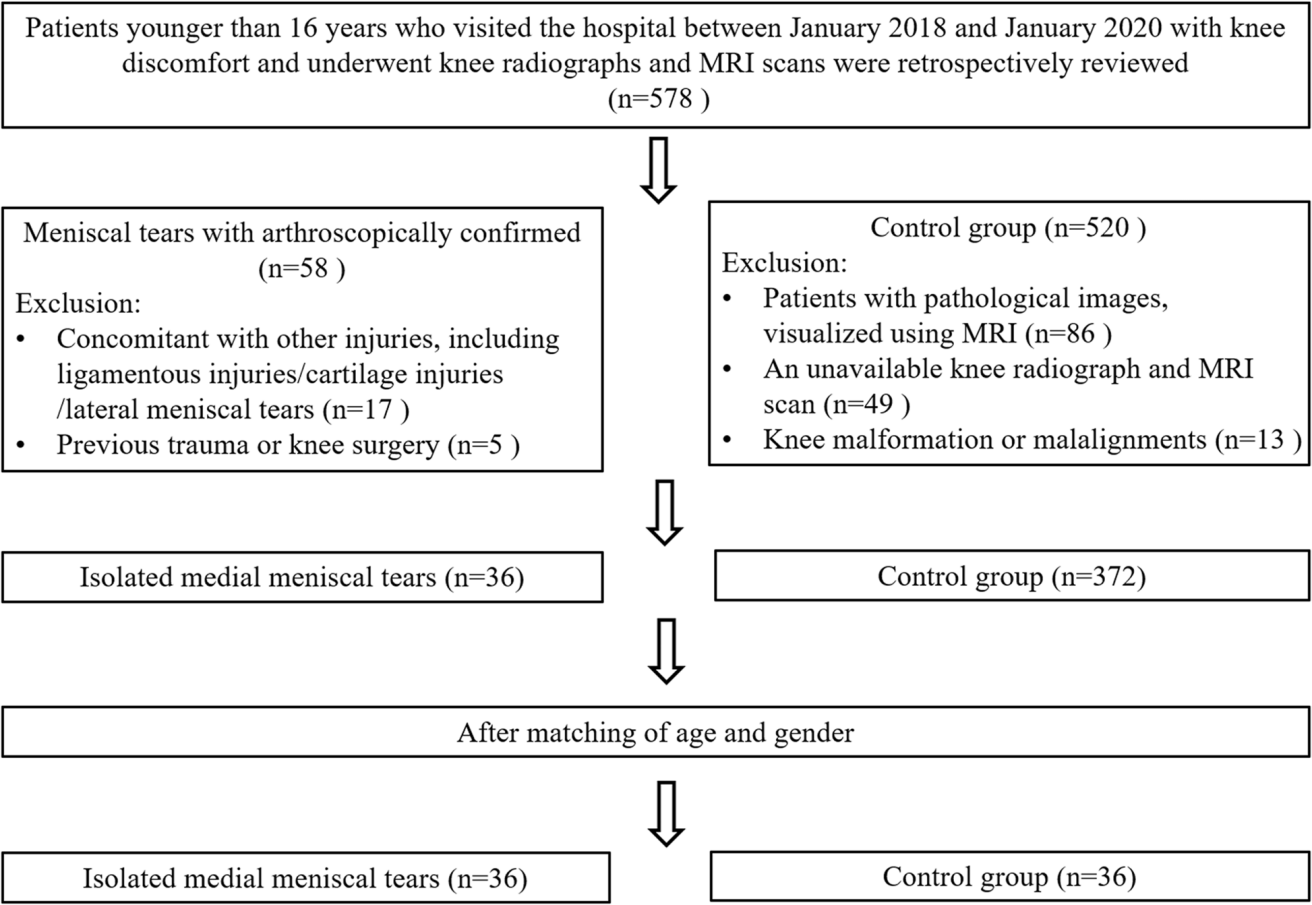
Flowchart illustrating the enrollment of patients for this study

Data from January 2018 to January 2020, patients who visited the hospital with knee discomfort and underwent knee radiographs and magnetic resonance imaging (MRI) scans were retrospectively reviewed, and they were divided into the two groups: isolated MMT group and control group, respectively. Inclusion criteria for isolated MMT group were as follows: a) patients younger than 16 years; b) presence of isolated MMT as visualized on MRI scan and reconfirmed with arthroscopy intraoperatively; c) good-quality of knee radiographs (with both femoral condyles completely overlapped in lateral views at approximately 30°of knee flexion) and MRI scans. Patients who met the following criteria were excluded: a) concomitant with other injuries, such as ligamentous injuries, severe cartilage defects, and lateral meniscal tears; b) patients with previous trauma or knee surgery affecting the bony anatomy of the knee joint. Moreover, the control group was defined as the knee MRI scans with normal radiological images during the same period. Patients were excluded from the control group if they had any pathological images, knee malformation or malalignment, and an unavailable knee radiographs or MRI. In this case-control study, the isolated MMT cases were matched by age and sex to a group of radiologically normal controls. Figure 1 shows the flow diagram of patient enrollment in the study.

### Radiological assessments

**Fig. 2:**
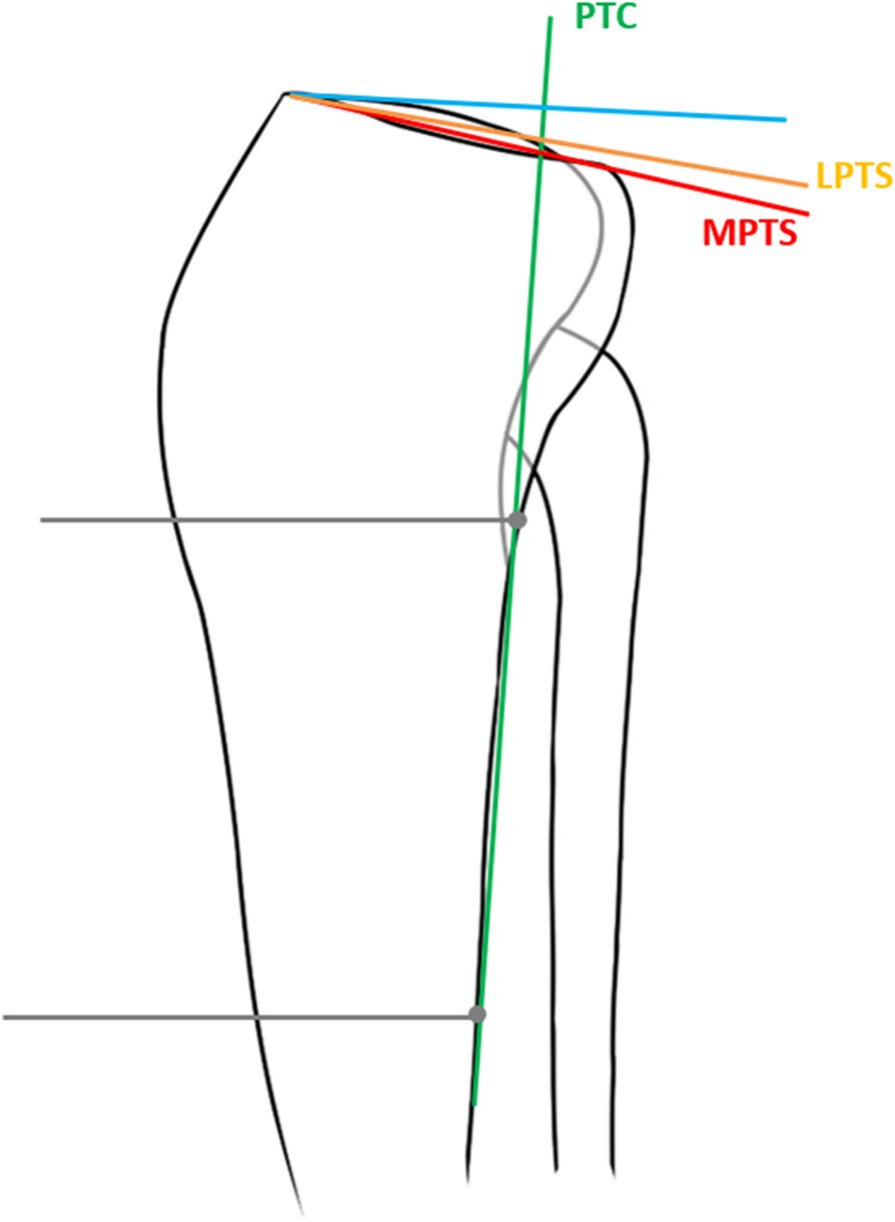
Schematic illustration showing the descried measurement technique for calculating medial and lateral tibial posterior tibial slope on lateral views. MPTS, medial posterior tibial slope; LPTS, lateral posterior tibial slope. PTC, proximal tibial cortex

**Fig. 3:**
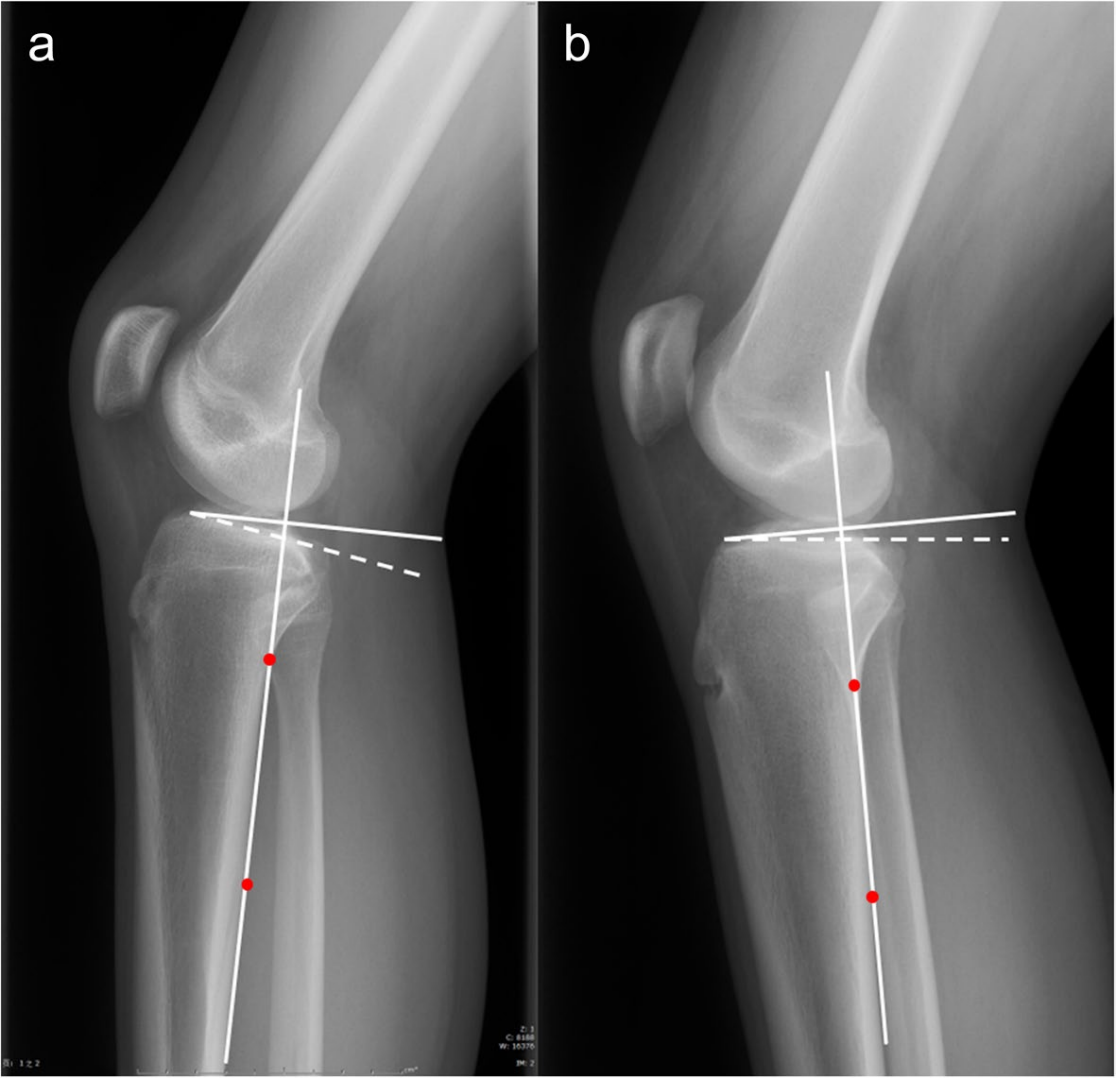
The PTS is defined as the angle between the dash line drawn along the anterior and posterior edges of the tibial plateau and the line perpendicular to the PTC. An example of the MPTS in (**a**) the MMT group (“actual degree”) and (**b**) control group (“actual degree”). MMT, medial meniscal tear; PTC, posterior tibial line; MPTS, medial posterior tibial slope

All patients included in the study underwent knee radiographs and MRI examination. There have been many techniques measuring PTS using different reference line in knee radiographs, and our measurement technique was based on the foundation of a previously validated method described by Hohmann et al. [20]. Two experienced musculoskeletal radiologists (Hu HZ, Song QC) who blinded to patients’ data independently assessed radiographic parameters twice using the measurement tools available in the picture archiving and communication system (PACS; Science & Technology General Company of the Third Hospital of Hebei Medical University, shijiazhuang, China) in our institution at an interval of 2 weeks to assess the intra- and inter-observer reliability. On lateral radiographic view both the medial/lateral posterior tibial slope (MPTS/LPTS) was defined as the angle between the perpendicular line to posterior cortical line (PTC) and the tangent line connecting the most superior points at the anterior and posterior edges of the respective tibial plateau, as shown in Figs. 2 and 3. The PTC was determined by two cortical points: the points located at 5- and 15- cm distal to the knee joint line [21], respectively. Once connecting these two points, the longitudinal axis of the PTC can be represented.

### Statistical analysis

SPSS software (version 24.0, IBM Corp., USA) was performed for statistical analysis. Continuous variables were tested for normality distribution by Kolmogorov–Smirnov test and then presented as mean ± standard deviation. After data normality was established, parametric variables were compared using Student’s *t*-test. The sample size was calculated according to a priori power analysis, and a sample size of 32 per group was considered as the minimum number for alpha of 0.05 and a power of 0.8. Receiver operating characteristic (ROC) curve analysis was performed to identify the optimal cut-off values of PTS. Also, the intra-observer and inter-observer reliabilities of PTS measurements were analyzed by intraclass correlation coefficients (ICCs). For all tests, statistical significance level was set at *p* < 0.05.

## Results

Twenty-two patients in the isolated MMT group were excluded due to ligamentous injuries, cartilage defects, lateral meniscal tears, and previous trauma or knee surgery. A total of seventy-two patients who met the inclusion and exclusion criteria were enrolled in the final analysis (36 with isolated MMT, and 36 controls). All cases in the MMT group sustained sprots-related meniscal tears. Each group consisted 21 males and 15 females. As shown in Table 1, the ICCs for the MPTS was 0.912 (95% CI, 0.875–0.954) for the intra-observer reliability and 0.894 (95% CI, 0.834–0.937) for the inter-observer reliability, and the ICCs for the LPTS was 0.878 (95% CI, 0.847–0.921) for the intra-observer reliability and 0.851 (95% CI, 0.804–0.913) for the inter-observer reliability, indicating an excellent inter- and intra-reliabilities.

**Table 1:** Intra-observer and inter-observer reliability among PTS measurements performed^a^

Variables	Intra-observer reliability ICC (95% CI)	Inter-observer reliability ICC (95% CI)
MPTS	0.912 (0.875–0.954)	0.894 (0.834–0.937)
LPTS	0.878 (0.847–0.921)	0.851 (0.804–0.913)

*ICC* intraclass correlation coefficient, *MPTS* medial posterior tibial slope, *LPTS* lateral posterior tibial slope

^a^ Values are presented as intraclass correlation (95% CI)

**Fig. 4:**
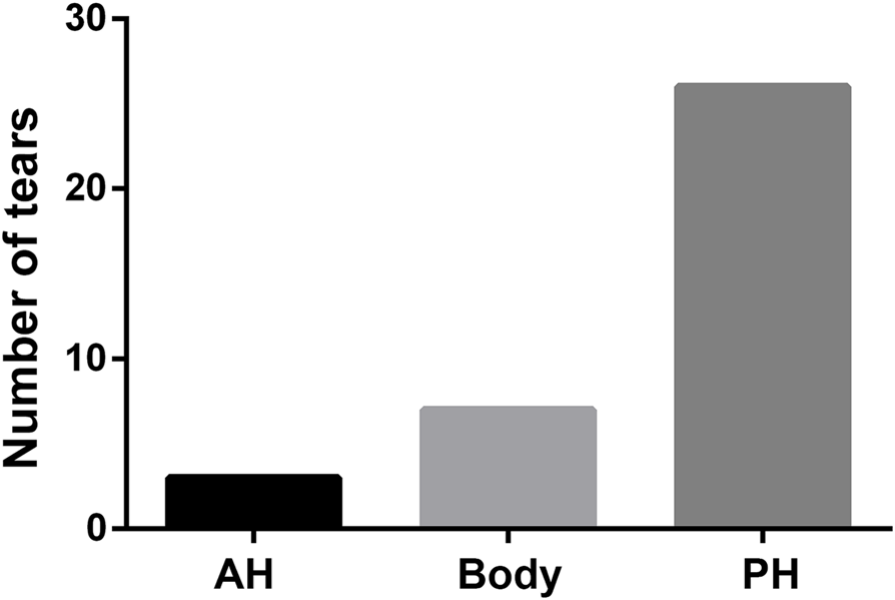
The distribution of meniscal tears across the different anatomical regions. AH, anterior horn; PH, posterior horn

**Fig. 5:**
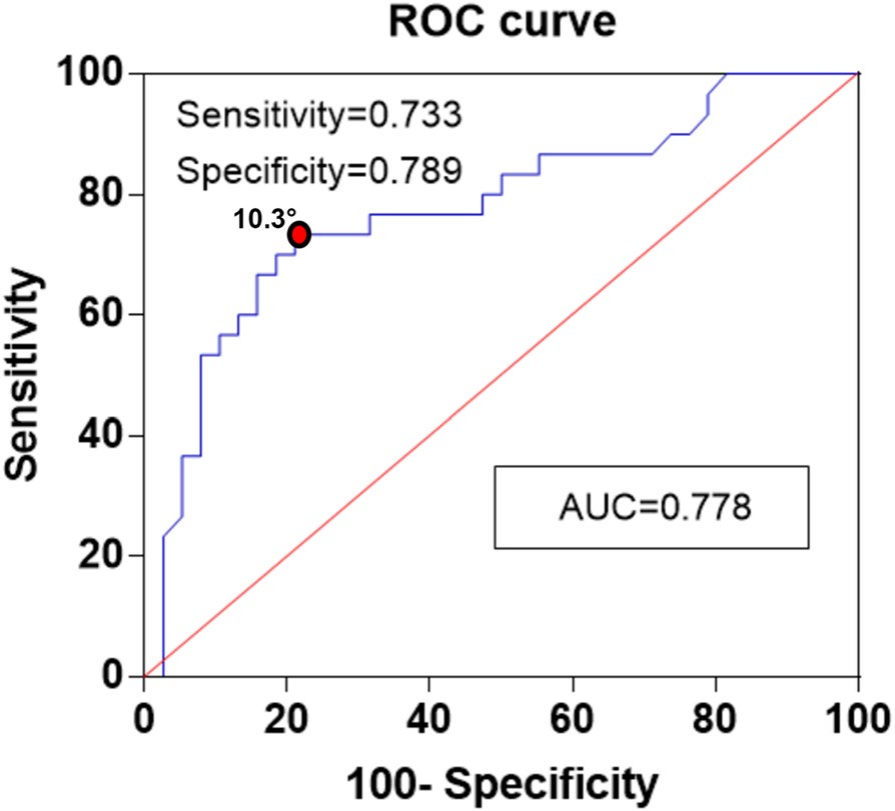
Receiver operating characteristic curve analysis for MPTS measurement. Reference line (diagonal): AUC =0.778. AUC, area under the curve; MPTS, medial posterior tibial slope

The patients’ demographic characteristics and PTS measurements of the two groups were shown in Table 2. No significant difference was observed between the two groups in terms of age (*p* = 0.566) and genders (*p* = 1.000). The MPTS was higher in the knees with isolated MMT than that of the controls (10.7° ± 2.1° vs 8.8° ± 1.7°, *p*<0.001). However, there was no significant difference regarding the LPTS between the isolated MMT and controls (11.5 ± 3.4 vs 10.9 ± 2.6, *p* = 0.403). To clarify the exact tear location of the MM, the meniscus was divided into three parts: the anterior horn (AH), the body, and the posterior horn (PH). In our study, the most common tear location of the MM was the posterior horn, followed by the tears involving the body. In addition, tears of the anterior parts of the menisci were seen least frequently. The distribution of meniscal tears in different anatomical location was shown in Fig. 4. According to the ROC analysis, the calculated optimal cut off value of MPTS with an increased risk for isolated MMT was 10.3°, with a sensitivity of 73.3% and specificity of 78.9% (Fig. 5).

**Table 2:** Patients’ demographic characteristics and measurements between MMT cases and healthy controls

	MMT group	Control group	*P* value
Patients	36	36	
Age (years)	15.0 ± 1.6	14.8 ± 1.2	0.566
Male/female	21/15	21/15	1.000
MPTS (°)	10.7 ± 2.1	8.8 ± 1.7	<0.001^*^
LPTS (°)	11.5 ± 3.4	10.9 ± 2.6	0.403

Control group, patients without any pathologic findings on MRI

Continuous variables are presented as mean ± standard deviation

*MMT* medial meniscal tears, *MPTS* medial posterior tibial slope, *LPTS* lateral posterior tibial slope

^*^Statistically significant (*P*<0.05)

## Discussion

The most important finding of the present study was that increased MPTS is associated with an increased risk for isolated MMT, whereas the LPTS appears to have less of an influence. These findings are similar with existing studies involving the adult population [22]. In addition, our results showed that the cut-off values of MPTS discrimination between the groups in adolescent patients was 10.3°, with a sensitivity of 73.3% and a specificity of 78.9%. This association may be originated from the biomechanical change and higher shear force in tibial plateau under the condition of a steep tibial slope. It has been reported that the tibial slope was an anatomical factor which have a great influence on the kinematics of tibiofemoral joint [23]. A large number of biomechanical studies have also shown a liner relationship between steep tibial slope and anterior translation of the tibia during weightbearing activities, which subsequently increased shear forces on ACL and meniscus [14, 24, 25]. Therefore, a steep tibial slope, leading to an increased anterior tibial translation, may predispose adolescent patients to MMT.

Actually, multiple literatures have shown that an association between increased PTS and meniscal tears in adults [11, 22, 26]. Kolbe et al. [11] identified that increased LPTS is a potential risk factor for posterolateral meniscal root tears in ACL-deficient knees. Moreover, Moon et al. [22] demonstrated that patients with medial meniscal posterior horn tears had significantly greater PTS than controls. Alici et al. [26] utilized both knee radiographs and MRI scans to measure PTS values in 212 meniscal tears patients, and they reported that the lateral meniscal tears group had increased LPTS when compared to the control group, whereas there is no association between the increased PTS values and MMT. These abovementioned published results provided some evidences that MMT are associated with increased tibial slope.

Despite the growing interests in PTS and its relationship with meniscal tears in adults [22, 27], this intrinsic anatomic factor remains unclear in adolescent patients. An increased demand to attain athletic activities and peak performance in adolescent patients may result in higher incidence of meniscal tears, especially in patients with increased tibial slope. The current study showed significant difference in MPTS values between the isolated MMT group and control group. Also, these findings were consistent with the results of Moon et al. [22], who demonstrated significant differences in MPTS between medial meniscal posterior horn tears patients and matched controls. Similarly, we assumed that this phenomenon may be partially explained by the function of medial meniscus, sustaining higher shear force and compressive loading forces in the presence of physiologic activities [28]. Therefore, anterior translation of the tibial that originate from a steep tibial slope may have a greater influence on medial meniscus. Of note, the main clinical significance of this study may have profound effects in the establishment of identification risk factors of MMT in a plain radiograph without using MRI.

The optimal cut-off value of MPTS was determined with the ROC curve analysis, which showed that the MPTS greater than 10.3° is associated with an increased risk for isolated MMT in adolescent patients. Recently, Moon et al. [22] identified that increased PTS (≥6.6°) was considered as an anatomical predictor for medial meniscal posterior horn tears with sensitivity of 55.3% and specificity of 75.0%. The reason for this discrepancy may be explained by the difference of patient selection. Dare et al. [29] revealed that PTS was tend to decrease or flatten with age, which may explain that the cut-off PTS values of the study was significantly higher than previously reported in adults. Another reason for this discrepancy may be attributed to the racial differences. Taken together, we considered that the adverse effect by increased tibial slope on MMT should not be overlooked in adolescents.

Several limitations should be noted in the present study. First, it was a retrospective study with the usual limitations and bias inherent to single center. To minimize bias, all observers who measured MPTS and LPTS on radiographs were blinded to the arthroscopic findings of meniscal status. Second, the number of patients enrolled in our study was relatively small and the location of meniscal tears were not evaluated in this study. However, the results of the presents study are meaningful in clinical significance, as the cut-off values of MPTS found in our study may be helpful for surgeons to consider intrinsic anatomical risk factors for MMT, especially with increasing number of meniscal tears in adolescents. Despite these limitations, there were important strengths associated with this study. To our knowledge, this is the first study to investigate the relationship between MPTS and MMT in adolescent patients. Also, these characteristics should be assessed by orthopedic surgeons using plain radiographs when suspect MMT in the adolescent patients.

## Conclusions

In conclusion, this study demonstrated that increased MPTS is associated with MMT, and MPTS≥10.3° was identified to be a risk factor for MMT in adolescents. This finding places an emphasis on the identification of risk factors for MMT, that is, if we recognize the steep MPTS in a plain radiograph, we can strongly suspect risk of MMT with using MRI.

## Data Availability

All the data and material involving this article will be available upon request by send an e-mail to the first author.

## Abbreviations

PTS: Posterior tibial slope
MPTS: Medial posterior tibial slope
LPTS: Lateral posterior tibial slope
MMT: Medial meniscal tears
MRI: Magnetic resonance imaging
ROC: Receiver operating characteristic curve
ACL: Anterior cruciate ligament
PTC: Proximal cortical line
PACS: Picture archiving and communication system
ICCs: Intraclass correlation coefficients
